# Abnormal histology in testis from prepubertal boys with monorchidism

**DOI:** 10.1186/s12610-020-00109-1

**Published:** 2020-08-06

**Authors:** Faruk Hadziselimovic, Gilvydas Verkauskas, Beata Vincel, Gunthild Krey, Zacharias Zachariou

**Affiliations:** 1 Institute for Cryptorchidism Research Liestal, Children’s Day Care Center Liestal, 4410 Liestal, Switzerland; 2 Children’s Surgery Centre, Faculty of Medicine, Vilnius University, Vilnius, Lithuania; 3 Medical School, University of Cyprus, Nicosia, Cyprus

**Keywords:** Monorchid testis, Histology, Immunohistochemistry, Fertility STAR, Testicule unique, Histologie, Immunohistochimie, Fertilité, Protein STAR

## Abstract

**Background:**

Little is known about the histology of contralateral descended testes in boys with unilaterally absent testis. We investigated whether absence of one testis is associated with abnormal tissue architecture of the solitary contralaterally descended testis.

**Design, setting, and patients:**

For this retrospective study, we examined the results of biopsies of the contralateral descended testis in 43 boys with monorchidism. Data from 26 control testes from boys of matching ages were selected from results published in 1977 and 2009. During surgery, any nubbins were removed. In each case, the scrotal testis was biopsied, and the testis fixed by subdartos pouch or suture.

**Results:**

Of the 43 affected boys, 23 had normal testicular histology in the contralateral descended testis, whereas 20 (46%) had abnormal histology. Eight of the abnormal biopsies matched the criteria for high infertility risk. Samples from three boys in this latter group revealed a Sertoli-cell-only phenotype. Immunohistochemical assays were positive for steroidogenic acute regulatory (STAR) protein in Leydig cells and spermatogonia. STAR expression was stronger in the monorchid group with normal testicular histology.

**Conclusions:**

Almost half of the patients with unilateral absent testis were at risk for subfertility or infertility. Our results emphasize the need for testicular biopsy of the solitary testis in boys with monorchidism to appropriately assess infertility risk.

## Résumé

**Contexte:**

Peux d’études ont analysé la structure des tissus testiculaires des testicules descendus controlatéraux chez les garçons avec des testicules unilatéraux absents. Nous avons investigué si l’absence congénitale d’un testicule est. associée à une histologie anormale des testicules descendus controlatéraux solitaires.

**Conception, contexte et patients:**

Cette étude rétrospective a examiné les résultats des biopsies des testicules descendus controlatéraux de 43 garçons monorchides. Les données de 26 testicules témoins ont été appariées surl’âge et sélectionnées à partir des données publiées en 1977 et 2009. Pendant l’opération, les nubins (reliquats) détectés ont été enlevés. Dans chaque cas, les testicules scrotaux ont fait l’objet d’une biopsie et d’une fixation par la technique de la valise ou par suture au subdartos.

**Résultats:**

Parmi 43 garçons, 23 avaient une histologie testiculaire normale dans les testicules descendus controlatéraux, tandis que 20 (46%) avaient une histologie anormale. Huit biopsies anormales correspondaient aux critères de risque élevé d’infertilité. Trois garçons de ce groupe avaient une histologie testiculaire montrant la présence de cellules Sertoli seules. L’analyse immunohistochimique de la protéine STAR a montré un signale dans les cellules de Leydig et dans les spermatogonies. L’expression STAR était plus forte dans le groupe des monorchides avec une histologie testiculaire normale.

**Conclusions:**

Près de la moitié des patients ayant des testicules congénitaux unilatéraux absents couraient un risque d’hypofertilité ou d’infertilité. Nos résultats soulignent la nécessité d’une biopsie testiculaire des testicules solitaires chez les garçons monorchides afin d’évaluer le risque d’infertilité de ces patients.

## Introduction

A unilaterally absent testis (monorchidism) is thought to be caused by prenatal or antenatal testicular torsion. The absent testis may be explained either by a twisted undescended gonad or twisted normal gonad that vanished because of events following complete descent. Therefore, vanishing testes and their descended counterparts may not be subject to the endocrinopathy that affects cryptorchid testes because torsion, the probable cause of monorchidism, is thought to occur along the path into the scrotum [1]. Of note, boys with cryptorchidism have a 10-fold higher incidence of testicular torsion compared to boys without cryptorchidism [2]. Histological examination of descended single testes in boys with a unilateral nubbin has revealed increased germ cell proliferation and dissimilar maturation patterns compared to the contralateral descended gonad in boys with unilateral cryptorchidism [3]. These findings point to antenatal torsion resulting from a mechanical event rather than endocrinopathy as the etiology for absent testis [3, 4]. The presence of a contralateral descended testis after nubbin excision raises concerns about future fertility. Normal histology of the monorchid testis [3] and normal paternity [5], as well as oligozoospermia [6] and azoospermia [7], have been observed in these patients. Here, we present histopathological results showing that half of the boys with monorchidism included in our study had pathologic testicular histology. This result is consistent with and may explain the increased incidence of subfertility or even infertility in males with unilateral absent testis.

## Patients and methods

Following approval by our institutional ethical committee, we conducted a retrospective database review that identified 43 boys who had undergone surgery for non-palpable testis. Inclusion criteria consisted of a preoperative diagnosis of a nonpalpable undescended testis and either the presence of a testicular nubbin or absent testis with blind-ending gonadal vessels and vas deferens at surgical exploration. During surgery, all testicular remnants (nubbins) detected were removed. Vanishing testes were confirmed during inguinal surgeries. In addition, the scrotal descended testis was biopsied in each case and fixed by subdartos pouch or suture.

Boys with monorchidism with or without a nubbin that completely lacked testicular tissue were included in the study. Investigated patients neither had chromosomal anomalies nor “re-do” surgeries performed earlier in life. Four boys with normal and 3 boys with abnormal testicular histology were hormonally treated prior to the surgery. All tissue samples were immediately fixed in 2% glutaraldehyde in phosphate buffer, embedded in epoxy resin, sectioned at a thickness of 1 μm, and stained with toluidine blue. Histological analysis was performed using light microscopy at 60× total magnification. For each biopsy, at least 100 seminiferous tubules were examined. Histological data included the total number of germ cells, the number of A dark (Ad) spermatogonia, and the presence of primary spermatocytes. Exclusion criteria were age > 18 years and missing data for any of the histological variables. Germ cell counts from the contralateral descended testis group and from a historical control group were compared. For the latter, data from 26 control testes, most of them obtained post-mortem, were selected from data published and age-matched [8, 9].

### Immunohistochemistry

16 testicular biopsies were analyzed (8 in each group). Fresh performed slides were blindly evaluated by two examiners experienced in immunohistology.

Immunohistochemistry was performed for steroidogenic acute regulatory (STAR) protein which is important in steroid hormone synthesis. It enhances the metabolism of cholesterol into pregnenolone by mediating the transfer of cholesterol to the inner mitochondrial membrane [10]. RNA-binding protein Sam68 belongs to the evolutionary conserved signal transduction and activation of RNA (STAR) family. It is required for the correct progression of spermatogenesis and for male fertility in the mouse and probably human [11]. Moreover, the decreased expression of Sam68 has been shown in human testes with spermatogenic defects demonstrating its role in the regulation of germ cells [12].

For immunohistochemical analysis, epon was removed from the tissue sections. The sections were treated with 2% bovine serum albumin to reduce non-specific binding and then incubated with the primary antibody overnight at 4 °C. All samples were washed with phosphate-buffered saline between incubations. We used an antibody targeting the steroidogenic acute regulatory (STAR) protein (Santa Cruz sc-166,821), labeled with horseradish peroxidase-polymer (HRP; goat polyclonal anti-rabbit IgG, mouse IgG and IgM, prediluted ab2891, Abcam, Cambridge, UK) to detect the binding of the primary antibody. The chromogenic reaction was developed by adding a freshly prepared solution of 3,3-diaminobenzidine solution (DAB + chromogen; DAKO). The DAB reaction was terminated by washing in Tris-buffered saline (0.05 M Tris-buffered saline and 0.85 M NaCl, pH 7.6). To allow visualization of testicular cells, the samples were counterstained with toluidine blue. Antibody binding was indicated by a brown precipitate. Different cell types were identified based on their nuclear morphology and position within the gonad. Immunohistochemistry experiments were performed at least three times on samples from at least eight patients from each group, and only those with identical results between experiments for each sample were included in the study. Controls for non-specific binding of the secondary antibody were performed in all experiments by omitting the primary antibody; these experiments consistently yielded no signal within the seminiferous epithelium or the interstitial space. Experimental design, biomaterials and treatments, reporters, staining, imaging data, and image characterization were performed in compliance with the minimum information specification for immunohistochemistry experiments [13].

### Statistical analysis

The software package StatXact 6.30 (2004, CYTEL Corporation) was used to apply the Mann–Whitney U test of unpaired data. In addition, Fisher’s exact test was applied, and *p* < 0.01 was considered to indicate significance.

## Results

Biopsy data were available for 43 prepubertal patients with absent unilateral testes. The mean total spermatogonia count was 2.3 ± 1.5 (95% confidence interval [CI] 1.8–2.8), and the mean Ad spermatogonia count for the whole group was 0.127 ± 0.129 (95% CI 0.008–0.16) per tubular cross section. Boys with monorchidism whose testis showed normal histology had a mean age of 2.79 ± 3.8 (95% CI 0.96–4.62) years at the time of surgery, which did not differ significantly from that of boys with monorchidism and abnormal testicular pathology (2.8 ± 1.85 [95% CI 1.58–4.08] years).

**Fig. 1:**
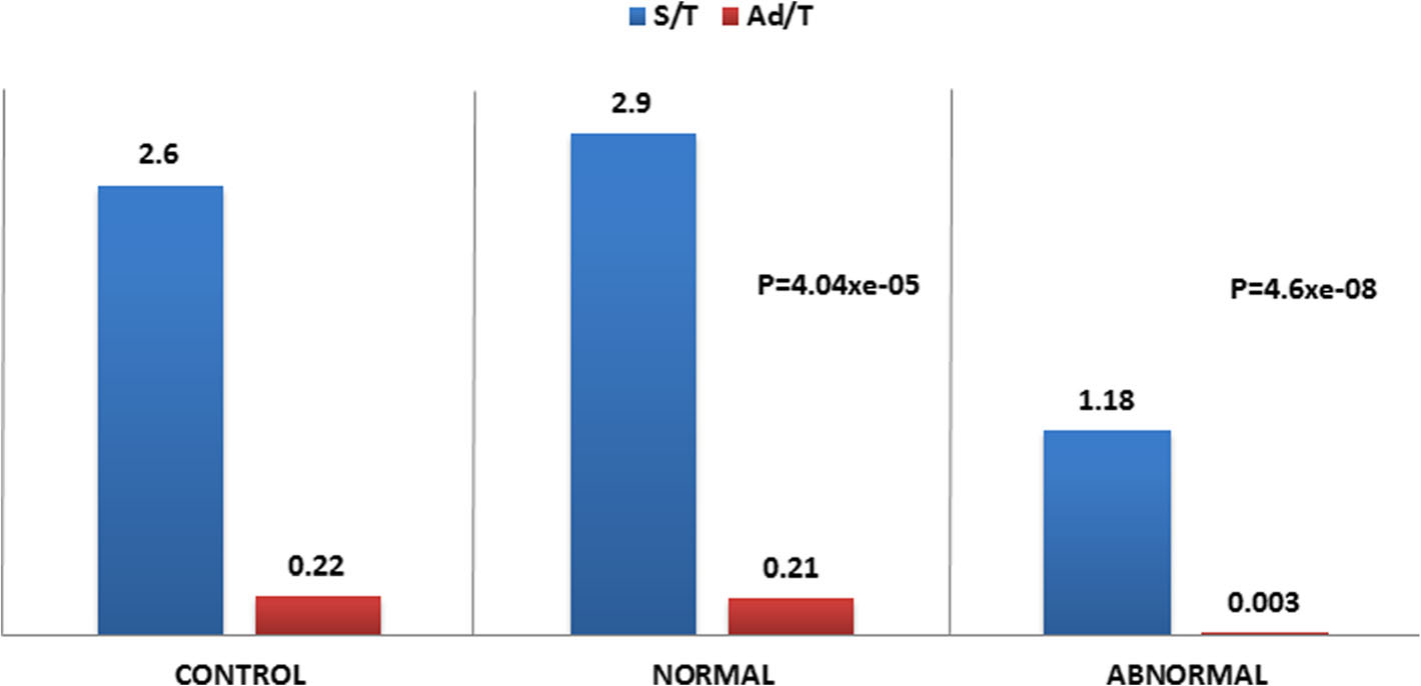
The total count (mean) of spermatogonia (S/T) and Ad spermatogonia (Ad/T) per tubular cross-section in both monorchid groups compared to the controls

Samples from 54% (*n* = 23) of the patients in our cohort showed normal testicular histology. The total mean count for germ cells was 2.9 ± 1.41 (95% CI 2.3–3.5) per tubule, whereas for Ad spermatogonia it was 0.21 ± 0.12 (95% CI 0.15–0.26) per tubule (Fig. 1). This count did not differ from that of the historical control group (Fig. 1).

In prepubertal testes, primary spermatocytes usually appear at 3–4 years of age [8]. In this study, in four of nine testes of boys with monorchidism, we observed spermatocytes at age 3–8 years (mean 0.24 ± 0.44 per tubular cross-section). This finding corresponds to a normal distribution in prepubertal boys with bilateral descended testes [8, 14]. Furthermore, none of the testes from the affected group displayed spermatogenesis that had progressed beyond primary spermatocytes, which would have indicated precocious puberty.

We did identify different degrees of testicular pathology in 46% (*n* = 20) of the affected boys. Mean values for both total germ cell count (1.18 ± 1.04 [95% CI 0.6–1.6] germ cells/tubule) and Ad spermatogonia (0.003 ± 0.002 [95% CI 0.0018–0.0035] per tubular cross-section) were reduced (Fig. 1). Samples from eight of the affected boys showed a testicular pathology consistent with a high risk of infertility. Three of these boys had Sertoli cells only, four had no Ad spermatogonia along with a reduced total germ cell count (> 0.2 < 0.6 germ cell/tubule), and one had no Ad spermatogonia and a total germ cell count of < 0.2 germ cell/tubule. Finally, 12 boys had intermediate or low risk of infertility.

**Fig. 2:**
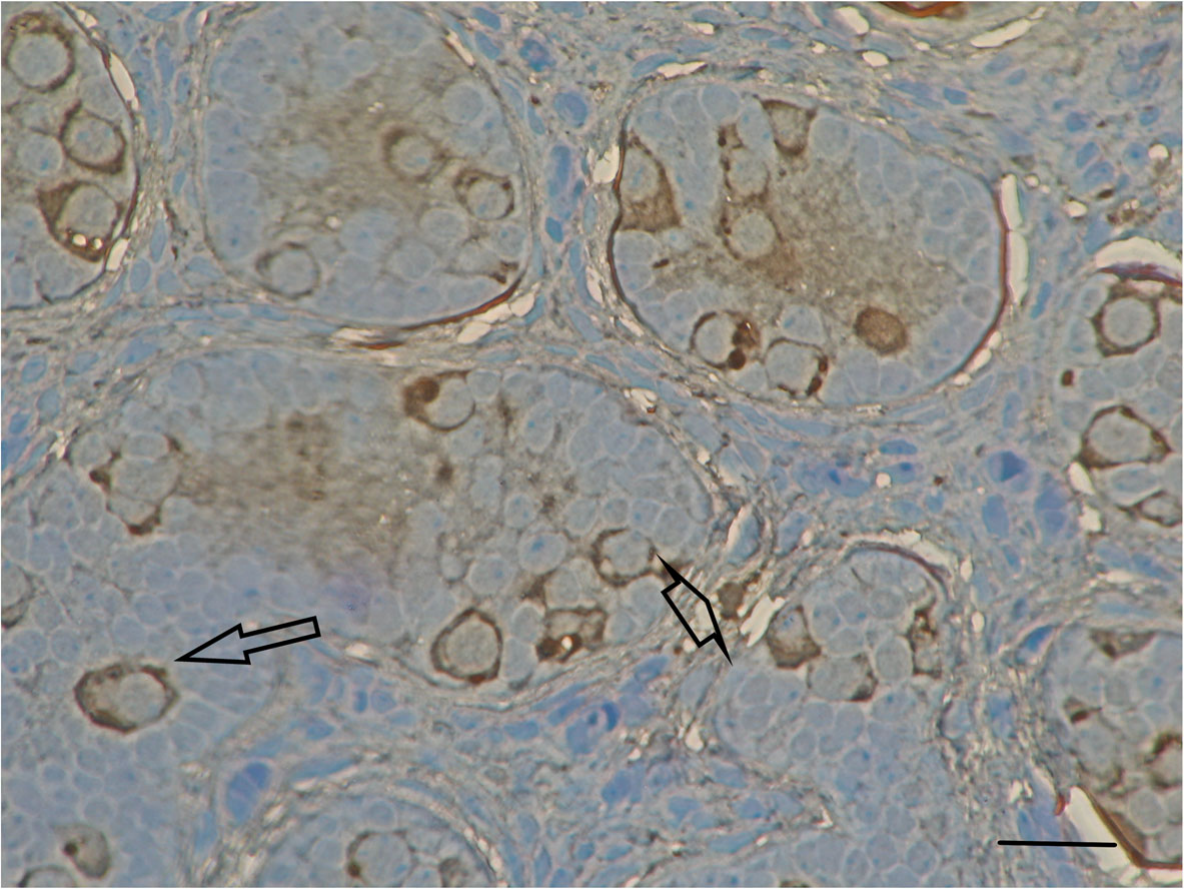
STAR protein staining in a sample from a 42-month-old boy with monorchidism and normal testicular histology. Intratubular spermatogonia (arrow) display strong STAR stain (4+) as well as the Leydig cells in interstitial tissue (arrowhead). Horizontal bar = 30 μ

**Fig. 3:**
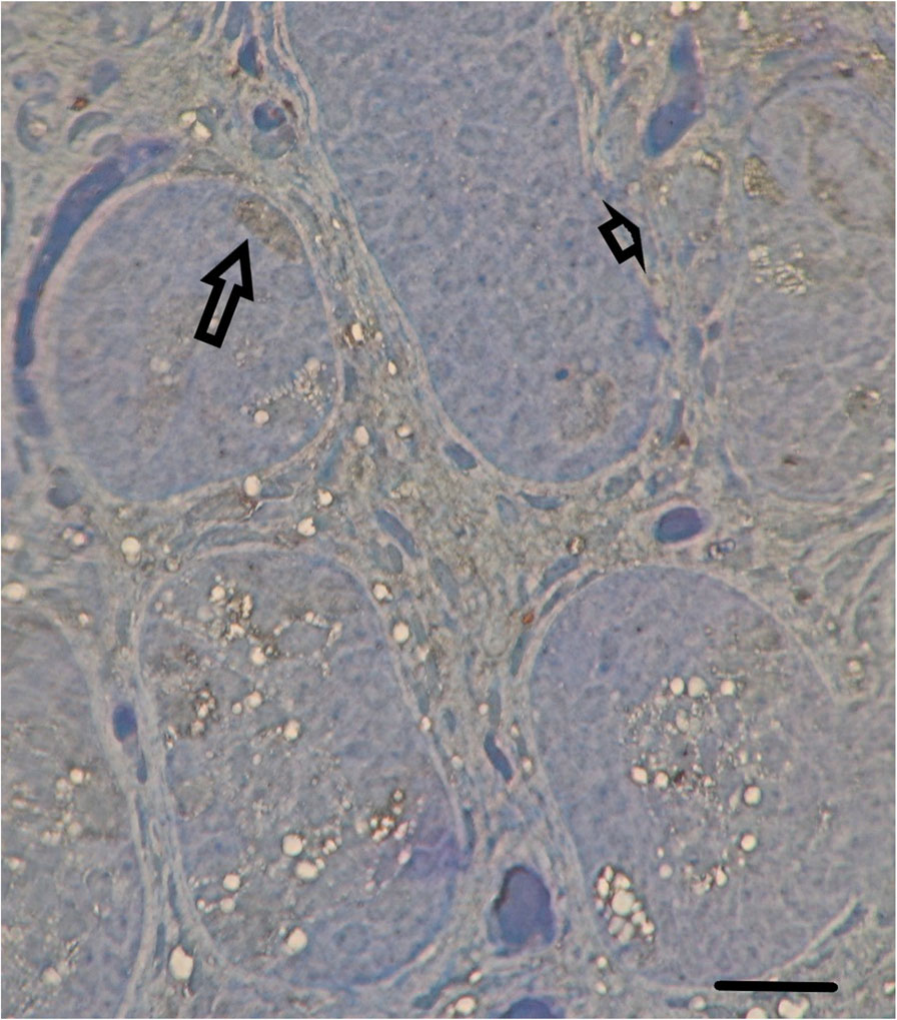
Weak staining for STAR protein (1+) from a 36-months-old boy at high risk for infertility: Leydig cells (arrowhead) located between the tubules and spermatogonia located at the basal membrane (arrow) are marked. Horizontal Bar = 30 μ

STAR immunoreactivity was observed exclusively in the cytoplasm in a granular pattern regardless of the tissue type studied. STAR was found in germ cells and Leydig cells. It was strongly expressed in testes with normal histology [4+] but only weakly so [1+] in testes with abnormal histology (Figs. 2 and 3).

## Discussion

In one study covering cases from 1985 to 1991, absent testes were diagnosed in 10% of 1225 patients examined for cryptorchidism [15]. Pathological studies of the remnants revealed a vas deferens in 89 cases (81%), epididymal tissue in 40 (36%), and small amounts of seminiferous tubules with germinal elements in seven (6.4%) [15]. A significant number of patients showed evidence of calcification (35.5%) and hemosiderin deposits (30%) within the remnant, lending weight to an etiology related to torsion rather than endocrinopathy [15]. Infants with monorchidism and contralateral testicular hypertrophy showed low inhibin B levels and high follicle-stimulating hormone levels, confirming the hypothesis that a single testis cannot prevent testicular insufficiency in adulthood [16]. In another study, STAR protein was detected in adult Leydig cells but not in adult spermatogonia [17]. In this context, we note that strong expression of STAR in prepubertal testicular germ cells has not been observed before (see Fig. 2). The presence of STAR in cells that do not express cytochrome P450 suggests that in addition to stimulating pregnenolone synthesis, STAR has roles in metabolic processes. However, the exact role of STAR in prepubertal germ cells is unknown. It may function in germ cell surveillance; testes with pronounced pathology and a decreased germ cell population also exhibited decreased STAR expression (Fig. 3).

The monorchid testis in boys with a unilateral testicular nubbin is generally believed to be normal and leave prospective fertility intact [3, 5]. Of note, Lee et al. did not find diminished paternity among men with a single testis compared to the general population, regardless of what led to the absent testis [5]. However, azoospermia has been reported in patients with monorchidism [7], and in one study, oligozoospermia was identified in 36% of 47 young males who had a single descended testis [6]. In that same study, the percentage of men with oligozoospermia did not differ between the 13 who had an absent testis (38.5%) and the 34 (35.3%) with an undescended testis [6].

\Few reports have described the quality of the testicular tissue of the contralateral descended testis from boys with a unilateral testicular nubbin [3, 4]. Kraft et al. described advanced testicular maturation in a group of boys with monorchidism [3]. In the current work, we found that none of the affected boys had testicular development that had advanced beyond spermatocytes, an indicator of advanced testicular maturation. In contrast to Kraft et al.’s results of normal or advanced testicular development among all monorchid testes, we found that half of the affected population in our study showed abnormal testicular histology. Of note, 9% of boys with monorchidism met the criteria for high risk of infertility, with a complete lack of Ad spermatogonia and a reduced total germ cell count [18, 19]. Theoretically, abnormal testicular histology could result from some adverse effects of the contralateral nubbin. A more likely hypothesis is that boys who are predisposed to develop infertility are more prone to testicular torsion. Boys with cryptorchidism, for example, are reported to have a higher incidence of testicular torsion along the path of descent compared to unaffected boys [2].

## Conclusions

In this study, we found that 46% of the patients with a congenitally absent testis on one side have an elevated risk of infertility. Therefore, boys with an absent testis should be examined by a contralateral testicular biopsy at the time of elective fixation of the solitary testis. The result will justify the application of a therapeutic regime to reduce the risk for impaired fertility in these boys. In cases in which a biopsy is not performed, the family should be advised that a sperm quality assessment should be sought for the patient in adulthood.

## Data Availability

Not applicable.

## Abbreviations

Ad: A dark spermatogonia
Ad/T: Number of Ad spermatogonia per tubular cross-section
STAR: Steroidogenic Acute Regulatory Protein
S/T: Number of spermatogonia per tubular cross-section
